# hsa-miR-203 enhances the sensitivity of leukemia cells to arsenic trioxide

**DOI:** 10.3892/etm.2013.981

**Published:** 2013-02-27

**Authors:** JIN-HUA HE, YU-MIN LI, YU-GUANG LI, XING-YI XIE, LI WANG, SHUN-YI CHUN, WU-JIA CHENG

**Affiliations:** 1 Department of Laboratory, Central Hospital of Panyu District, Guangzhou, Guangdong 511400;; 2 Department of Clinical Laboratory, Minzu Hospital, Nanning, Guangxi 530001, P.R. China

**Keywords:** hsa-miR-203, chronic myelogenous leukemia, arsenic trioxide, apoptosis, eukaryotic expression vector

## Abstract

The aim of this study was to investigate the effect of a eukaryotic expression vector expressing hsa-miR-203 on the sensitivity of K562 leukemia cells to arsenic trioxide (ATO) and the possible mechanism of action. The eukaryotic expression vector expressing the hsa-miR-203 plasmid (PmiR-203) was transfected into K562 cells using Lipofectamine 2000. bcr/abl 3′ untranslated region (UTR) and bcr/abl mutated 3′UTR dual luciferase report vectors (psi-CHECK-2) were used to validate the regulation of bcr/abl by miR-203. The inhibitory effects of ATO and PmiR-203, used singly or in combination, on cell proliferation were detected by MTT assay. Apoptosis of the K562 cells was detected by flow cytometry using double-staining with Annexin V and propidium iodide (PI). The activities of caspase-3 and caspase-9 were detected by a colorimetric method and the cytochrome *c* protein levels were detected by western blotting. When used in combination with PmiR-203, the IC_50_ of ATO was reduced from 6.49 to 2.45 *μ*g/ml and the sensitivity of cells to ATO increased 2.64-fold. In addition, PmiR-203 and ATO caused growth inhibition, apoptosis and G1-phase arrest in K562 cells. Furthermore, PmiR-203 significantly promoted ATO-mediated growth inhibition and apoptosis, affecting the G1 phase. JC-1 fluorescent staining revealed that the membrane potential of the mitochondria had changed. The activities of caspase-3 and caspase-9 increased, the expression levels of cytochrome *c* were upregulated and the expression level of bcr/abl mRNA was significantly suppressed. Furthermore, the dual-luciferase reporter vector, containing tandem miR-203 binding sites from the bcr/abl 3′UTR, demonstrated that bcr/abl was directly regulated by miR-203. PmiR-203 sensitized K562 leukemia cells to ATO by inducing apoptosis and downregulating bcr/ abl gene levels. The induction of apoptosis may occur through the mitochondrial pathway. The combination of ATO and PmiR-203 presents therapeutic potential for chronic myelogenous leukemia.

## Introduction

With >500 microRNA (miRNA) genes identified experimentally in the human genome and a plethora of computationally-predicted mRNA targets, it is believed that these small RNAs have a central role in diverse cellular and developmental processes. Therefore, the aberrant expression of miRNA genes may lead to human disease, including cancer. Several studies have confirmed that miRNAs regulate cell proliferation and apoptosis (1–3). Arsenic trioxide (ATO), an ancient traditional Chinese medicine, has been successfully used as a therapeutic agent for leukemia (4). Drug resistance and toxicity are major concerns with this treatment. miRNAs are endogenous small non-coding RNA molecules that may modulate cellular sensitivity to anticancer drugs (5). miR-203 is overexpressed in pancreatic adenocarcinoma cells and demonstrates a correlation with poor prognosis in patients that have undergone pancreatectomy. It has also been proposed as a tumor-suppressive miRNA in hepatocellular carcinoma (6,7). However, miR-203 has been rarely characterized in chronic myelogenous leukemia (CML). In our previous study, a eukaryotic expression vector carrying the hsa-miR-203 gene was successfully constructed and PmiR-203 was able to effectively inhibit the proliferation and promote the apoptosis of K562 cells (8). Therefore, we questioned whether miR-203 modulates the chemosensitivity of leukemia cells. In the present study, we evaluated the role of PmiR-203 and its effect on ATO treatment. We aimed to provide mechanistic evidence for the synergistic effects of PmiR-203 and ATO in K562 leukemia cells. Consequently, the potential of miR-203 should be explored as a gene therapy for the treatment of leukemia.

## Materials and methods

### Materials

Fetal calf serum (FCS) and Dulbecco’s modified Eagle’s medium (DMEM)/F12 were obtained from Invitrogen Life Technologies (Carlsbad, CA, USA). *Not*I, *Xho*I (Takara Bio Inc., Shiga, Japan), T4 DNA Ligase (Takara Bio Inc.), RNasin inhibitor (Promega Corporation, Madison, WI, USA), Hoechst 33258, 3-(4,5-dimethylthiazol-2-yl)-2,5-diphenyltetrazolium bromide (MTT), Annexin V-fluorescein isothiocyanate (FITC) and propidium iodide (PI; Sigma, St. Louis, MO, USA) were also used in this study.

### Design and construction of the eukaryotic expression vector expressing hsa-miR-203

The mature miRNA-203 sequences are available from the miRNA Registry (5′-GTGAAATGTTTAGGACCACTAG-3′). In order to prevent the formation of a termination signal, TTGGCCACTGACT was selected as the region in a miRNA expression vector template. TGCT was added to the 5′ positive-sense strand template of the miRNA expression vector and GTCC was added to the 5′ antisense strand template. Further, a non-specific sequence was designed and sent to GenePharma Co., Ltd. (Shanghai, China) for synthesis and finally, a eukaryotic hsa-miR-203 expression vector was successfully constructed. The assay was performed as previously described (8).

### Cell line and DNA transfection

The K562 (CML) cell line (Shanghai Institute of Cell Biology, China) was grown in DMEM/F12 containing 10% FCS at 37°C in a humidified atmosphere with 5% CO_2_ in a Thermo FORMA 3110 incubator (Thermo Scientific, Marietta, OH, USA). K562 cells in the exponential phase of growth were seeded in 96- or 24-well plates (Costar, Cambridge, MA, USA) and transfected with the plasmid using Lipofectamine 2000 reagent (Invitrogen; 1:1.2 volume/mass ratio of Lipofectamine 2000 to the plasmid) in serum-free DMEM/F12 for 6 h. At the end of transfection, the cells were incubated in medium containing 10% FCS. Transfection of PmiR-203 into the K562 cells with a final concentration of 500 ng/*μ*l was performed according to the manufacturer’s instructions.

### Plasmid construction and site-directed mutagenesis

The 3′ untranslated regions (3′-UTRs) of the bcr/abl genes were amplified by polymerase chain reaction (PCR) using genomic DNA of the K562 cell line and cloned downstream of the Renilla luciferase open reading frame in the psiCHECK-2 vector (Promega) using *Xho*I and *Not*I restriction sites. The bcr/abl primers (CCGCTCGAGCAGCAGTCAGGGGTCAGG and ATAAGAATGCGGCCGCTTCTAATGTAAACACTG ATTTATTTA) were designed to bind to position 1992 of the bcr/abl genomic sequence. Tandem mutations were introduced to the seed region of the miR-203 binding site in the primers; UGUAAAGU was substituted by AUCGAUC and the construct was named bcr/abl-mut-UTR.

### Luciferase reporter assay

For luciferase reporter assays, cells were seeded in 96-well plates and cotransfected with 25 nM miRNA mimics or 100 nM hairpin inhibitors together with 15 ng/well psiCHECK-2 reporter vectors. Forty-eight hours after transfection, luciferase activity was measured using the dual-luciferase reporter assay system kit (Promega) according to the manufacturer’s instructions and a Tecan M200 luminescence reader (Tecan Group Ltd., Männedorf, Switzerland). Values were double normalized to firefly luciferase activity and to cells transfected with empty psiCHECK-2 control vectors. The sequenc inclued Bcr/abl 3′UTR, 5′-UCUGAGUUCUUGAAGCAUUUCAA-3′, hsa-miR-203, 3′-GAUCACCAGGAUUUGUAAAGUG-5′ and Bcr/abl-mut 3′UTR, 5′-UCUGAGUUCUUGAAGAUCGAUCA-3′.

### ATO sensitivity assay

The viability of K562 cells was determined by the MTT assay. Briefly, cells at a density of 5×10^4^ cells/ ml were transfected with PmiR-203 or a negative control (NC; plasmid containing a scrambled sequence, 0.8 *μ*g/ml) in the presence of Lipofectamine 2000 and serum-free DMEM/F12 media for 6 h. The cells were plated in 96-well plates in medium containing 10% FCS for another 48 h, with the presence of varying concentrations of ATO (1.25, 2.5, 5.0, 10.0 and 20.0 *μ*g/ml). Then, 20 *μ*l MTT stock solution (5 mg/ml) was added to each well to a final MTT concentration of 0.45 mg/ml and the plate was incubated for 4 h at 37°C. The medium was then removed and dimethyl sulfoxide (DMSO; 150 *μ*l) was added to dissolve the blue formazan crystals at room temperature for 30 min. The viability of the cells was assessed by absorbance at 570 nm on a Bio-Rad microtiter plate reader (Hercules, CA, USA). The IC_50_ values were determined using IC_50_ software (Northwest A & F University, Beijing, China).

### Hoechst 33258 staining

K562 cells transfected with PmiR-203 (0.8 *μ*g/ml) were incubated for 48 h. Additionally, K562 cells treated with ATO (10 *μ*g/ml) were also grown for 48 h. Then, the cells were collected by centrifuging at 4°C for 6 min at 111.8 × g and washed in phosphate-buffered saline (PBS). The cells were sprayed onto glass slides, air-dried and fixed with methanol for 10 min. Then, the cells were washed with buffer twice, stained with Hoechst 33258 for 10 min, washed with distilled water and finally air-dried again. The morphology of the K562 cells was observed under a fluorescent microscope.

### Detection of mitochondrial membrane potential

K562 human CML cells were incubated in 12-well flat-bottomed plates at a concentration of 1.0×10^6^ cells/ml. Experimental groups: 1, control group with blank cells (control); 2, negative control (NC, containing plasmid, Scramble sequence 0.8 *μ*g/ml); 3, PmiR-203; 4, ATO. K562 cells transfected with PmiR-203 (0.8 *μ*g/ml) were incubated for 48 h. Meanwhile, K562 cells treated with ATO (10 *μ*g/ml) were also grown for 48 h. The cells were collected by centrifugation at 4°C for 6 min at 111.8 × g and washed in PBS. The cells were sprayed on glass slides, air-dried and fixed with methanol for 10 min, and then washed with buffer twice, stained with Hoechst 33258 stain for 10 min, washed with distilled water, and finally air dried. The morphology of the K562 cells was observed under a fluorescent microscope. After cultivation for 48 h, the cells were collected and washed with PBS. They were then suspended in 500 *μ*l JC-1 culture fluid, incubated in a 5% CO_2_ atmosphere at 37°C for 20 min and washed twice with incubation buffer. The cells were then suspended in 500 *μ*l incubation buffer. A drop of the cell suspension was placed on a microscope slide, covered with a cover glass and then observed under a fluorescence microscope.

### Flow cytometric analysis of the cell cycle and apoptosis

K562 cells were seeded at a density of 1.0×10^5^ cells/ml (500 *μ*l/well) in 24-well plates (Costar) and transfected with miR-203 (0.8 *μ*g/ml) using Lipofectamine 2000 reagent in serum-free DMEM/F12 for 6 h. Following transfection, 500 *μ*l appropriate growth medium containing 20% FCS was added to each well with a total volume of 1,000 *μ*l. Cells were continuously incubated for another 48 h in the presence of 10.0 mg/ml ATO, then harvested, washed twice with PBS and fixed with 70% ethanol. They were also treated with RNase A (1 mg/ml) following the elimination of ethanol. Finally, the cells were stained with PI solution (50 *μ*g/ml). The cell cycles were analyzed by flow cytometry according to the content of DNA (Beckman Coulter Elite, Fullerton, CA, USA). Pretreatment of the K562 cells was performed as described above. Viable cells were collected and double-stained with FITC-conjugated Annexin V and PI. For each sample, data from ∼10,000 cells were recorded in list mode on logarithmic scales. Apoptosis and necrosis were analyzed by quadrant statistics on PI-negative, Annexin V positive and PI and Annexin V-positive cells.

### Detection of caspase-3 and caspase-9 activity

Pretreatment of K562 cells was performed as follows: 1, control group with blank cells (control); 2, negative control (NC,containing plasmid, Scramble sequence 0.8 *μ*g/ml); 3, PmiR-203 (0.8 *μ*g/ml); 4, ATO (10 *μ*g/ml); 5, PmiR-203+ATO. The total RNA from treated cells was extracted in Trizol (Invitrogen) and was quantified by an ultraviolet spectrophotometer (UVP, Upland, CA, USA) at a wavelength of 260 nm. The Bcr/abl expression level was determined by real-time PCR. Reverse transcribed with an M-MLV reverse transcriptase. A 20 *μ*l reverse transcription (RT) reaction was incubated at 37°C for 30 min. Real-time PCR was performed using a PCR amplifier (Bio-Rad). The PCR cycles were as follows. There was an initial denaturation at 94°C for 3 min. The reaction was repeated for 40 cycles; each cycle consisted of denaturing at 94°C for 20 sec, annealing at 50°C for 25 sec and synthesis at 72°C for 20 sec, according to the manufacturer’s instructions. The GAPDH group was used as the internal control. The expression level was calculated using CT and 2^−ΔΔCt^. Then, the cells were lysed with RIPA buffer in the presence of a proteinase inhibitor (Shenergy Biocolor BioScience and Technology Co., Ltd., Shanghai, China). The protein concentration was determined using bicinchoninic acid (BCA; Bioss, Beijing, China) and adjusted to 2.0 *μ*g/*μ*l. Then, 50 *μ*l cell suspension containing 100 *μ*g proteins was extracted from each well. Lysis buffer (50 *μ*l) was used as the control. Next, 0.5 *μ*l dithiothreitol (DTT) was added for each 50 *μ*l 2X reaction buffer and 50 *μ*l prepared 2X reaction buffer was added to each well. Additionally, 5 *μ*l caspase-3 substrate or caspase-9 substrate was added to the wells. The plates were incubated in the dark at 37°C for 4 h and the A405 value was detected using an enzyme-labeled instrument (Bio-Tek, Winooski, VT, USA).

### Analysis of bcr/abl mRNA levels

Pretreatment of K562 cells was performed as described above. The total RNA from the treated cells was extracted using TRIzol (Invitrogen) and was quantified using an ultraviolet spectrophotometer (UVP, Upland, CA, USA) at a wavelength of 260 nm. The bcr/abl expression level was determined by real-time PCR using M-MLV reverse transcriptase. A 20 *μ*l reverse transcription reaction was incubated at 37°C for 30 min. Real-time PCR was performed using a PCR amplifier (Bio-Rad). The PCR cycles were as follows: initial denaturation at 94°C for 3 min, then 40 cycles of denaturing at 94°C for 20 sec, annealing at 50°C for 25 sec and synthesis at 72°C for 20 sec, as per the manufacturer’s instructions. The GAPDH gene was used as the internal control. The expression level was calculated using cycle threshold (Ct) values and the 2^−ΔΔCt^ method.

### Western blot analysis of cytochrome c protein levels

The cells were analyzed in RIPA buffer in the presence of a proteinase inhibitor. The protein concentration was determined using BCA. Proteins (30 *μ*g) were separated using 10% sodium dodecyl sulfate-polyacrylamide gel electrophoresis (SDS-PAGE) and transferred to a nitrocellulose membrane. Membranes were probed with primary antibodies against cytochrome *c* (rabbit polyclonal; Cell Biotech, Tianjin, China) at room temperature for 2 h, washed extensively with 0.1% Tween-20 in PBS and incubated with secondary antibodies conjugated with horseradish peroxidase at a dilution of 1:10,000 (Pharmingen, Becton Dickinson, San Diego, California, USA), at room temperature for 3 h. Development was performed using an enhanced chemiluminescence (ECL) system (Amersham Pharmacia Biotech, Amersham, UK).

### Statistical analysis

All results and data were confirmed in at least three separate experiments. Data are expressed as mean ± standard deviation (SD). Statistical comparisons were made by one-way analysis of variance (ANOVA). P<0.05 was considered to indicate a statistically significant difference.

## Results

### bcr/abl is directly regulated by miR-203

**Figure 1:**
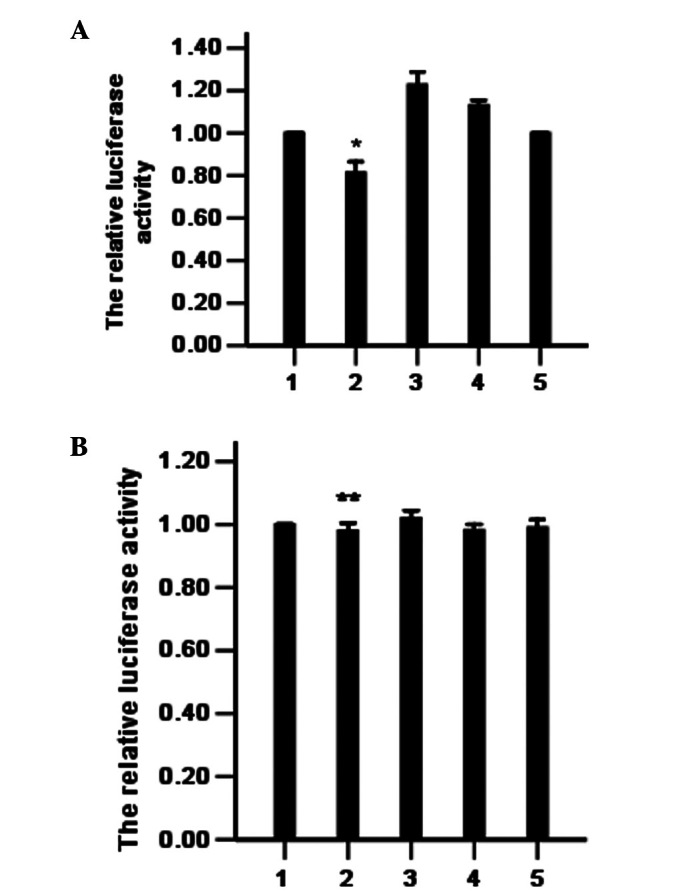
Interaction between miR-203 and bcr/abl was detected by luciferase reporter assay. (A) Luciferase activity comparison for a plasmid transfected with the bcr/abl-3′untranslated region (UTR). ^*^P<0.05 vs. control and NC groups. (B) Luciferase activity comparison for a plasmid transfected with bcr/abl-mut-3′UTR. ^**^P>0.050 vs. control and NC groups. 1, Control group; 2, miR-203 mimic group; 3, inhibitor group: 4, NC group; 5, NCI group. NC, negative control; NCI, negative control with inhibitor.

To confirm bcr/abl as a direct target of miR-203 in K562 leukemia cells, we constructed the dual-luciferase reporter (psi-CHECK) containing either the miR-203 recognition sequence or a mutated sequence from the 3′UTR of bcr/abl mRNA immediately downstream of the luciferase gene. As shown in Fig. 1, transfection with miR-203 reduced the activity of the bcr/abl-UTR reporter in the K562 cells but did not affect the activity of the bcr/abl-mut-UTR reporter. These results suggest that bcr/abl is a target of miR-203 in K562 cells.

### PmiR-203 promotes ATO sensitivity in leukemia cells

**Figure 2:**
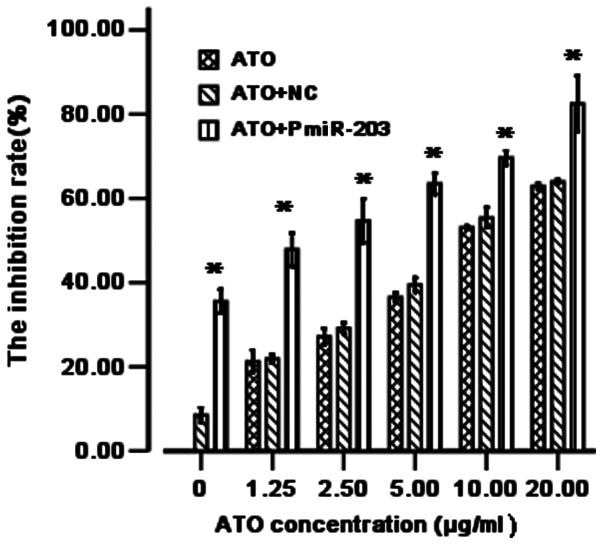
PmiR-203 promotes the sensitivity of K562 cells to arsenic trioxide (ATO). K562 cells were transfected with PmiR-203 or a negative control (0.8 *μ*g/ml) in the presence of Lipofectamine 2000 and serum-free Dulbecco’s modified Eagle’s medium (DMEM)/F12 for 6 h. At the end of transfection, the cells were plated in 96-well plates in medium containing 10% fetal calf serum (FCS) for another 48 h in the presence of varying concentrations of ATO (1.25, 2.5, 5.0, 10.0 and 20.0 *μ*g/ml). Cell viability was assessed by MTT assays and performed in triplicate. The results indicated that PmiR-203 inhibits K562 cell viability and promotes ATO-induced growth inhibition. ^*^P<0.05 compared with the NC+ATO group and the ATO group. NC, negative control.

In this study, we determined the effect of PmiR-203 on cell viability (alone or in combination with ATO). As shown in Fig. 2, PmiR-203 alone effectively inhibited cell viability. The data indicate that PmiR-203 is also able to increase the ATO-induced inhibitory effects on K562 cells. Thus, PmiR-203 significantly decreases the IC_50_ values of ATO. When used alone, the IC_50_ of ATO was 6.49 *μ*g/ml and when used in combination with the NC, the IC_50_ of ATO was 5.8 *μ*g/ml. However, when used in combination with PmiR-203, the IC_50_ of ATO was 2.45 *μ*g/ml. The susceptibility of the cells increased 2.64-fold (Fig. 2).

### Cell morphology validates cell apoptosis

**Figure 3:**
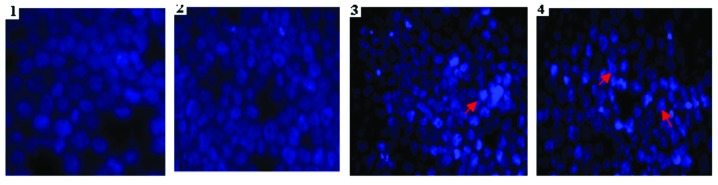
K562 cells were stained with Hoechst 33258 for 15 min at 48 h post-transfection. The morphologic aspects of the nuclei were observed under a light microscope. Arrows indicate apoptotic bodies. 1, Control group; 2, NC group; 3, PmiR-203; 4, arsenic trioxide (ATO). NC, negative control.

Hoechst 33258 staining (Fig. 3) also revealed that K562 cells in the PmiR-203 and ATO groups acquired typical features of apoptosis, including cell shrinkage, nuclear pyknosis and apoptotic bodies at 48 h post-transfection. The results confirmed that PmiR-203 and ATO are able to induce cell apoptosis.

### Detection of the mitochondrial membrane potential

**Figure 4:**
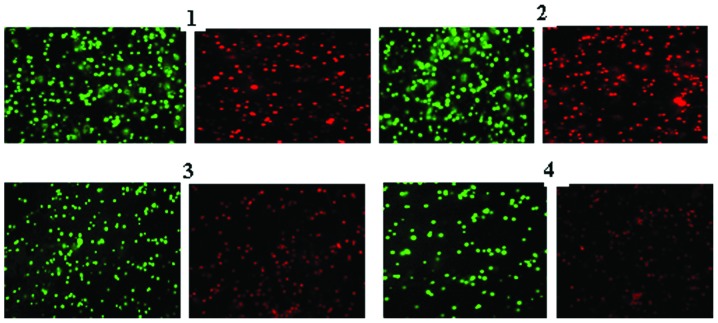
K562 cells were stained with JC-1 for 15 min at 48 h post-transfection. The changes in the mitochondrial membrane potential were observed under a light microscope. PmiR-203 and arsenic trioxide (ATO) were able to independently induce apoptosis, which is presented as high-green and low-red fluorescence. 1, Control group; 2, NC group; 3, PmiR-203; 4, ATO. NC, negative control.

Under a bicolor filter, while normal cells show high-intensity green and red fluorescence, apoptotic cells show high-intensity green fluorescence and low-intensity red fluorescence. The results of the negative control group were similar to those of the cell control group. All the cells in the former group demonstrated high-intensity green and red fluorescence, while the cells in the PmiR-203+ATO group demonstrated high-intensity green fluorescence and low-intensity red fluorescence (Fig. 4).

### PmiR-203 regulates the cell cycle as determined by flow cytometry

**Figure 5:**
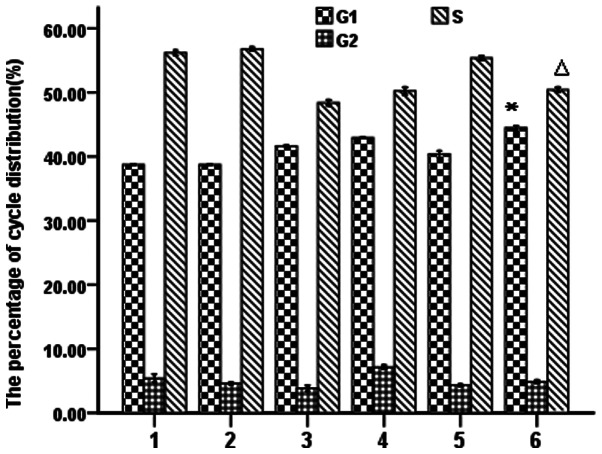
PmiR-203 affects the cell cycle in K562 cells. Cell cycles were analyzed by flow cytometry at 48 h post-transfection. The results indicated that miR-203 and arsenic trioxide (ATO) were able to increase the G1 phase of the cells. miR-203 promoted ATO-induced G1 phase cells. ^*^P<0.05 vs. the ATO and PmiR-203 groups; ^Δ^P>0.05 vs. the ATO group. 1, Control; 2, negative control (NC); 3, PmiR-203; 4, ATO; 5, ATO+NC; 6, PmiR-203+ATO.

To explore the effects of PmiR-203 on the cell cycle, PmiR-203 treatment alone or in combination with 10.0 *μ*g/ml ATO was investigated in K562 cells. Cells were stained with PI solution. Cell cycles were analyzed by flow cytometry according to the DNA content. As shown in Fig. 5, PmiR-203 and ATO were able to independently induce G1 phase arrest. The increased sub-G1-phase cell population indicates that PmiR-203 and ATO induce apoptosis in K562 cells (Fig. 5).

### PmiR-203 induces cell apoptosis as determined by flow cytometry.

**Figure 6:**
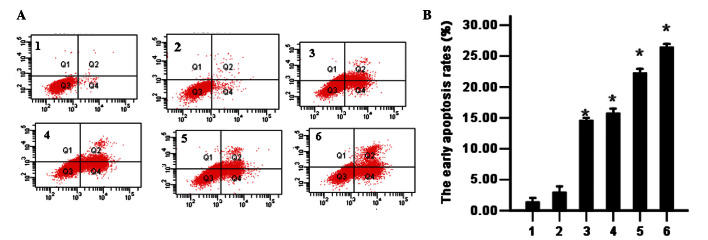
PmiR-203 affects the apoptosis of K562 cells. Viable cells were collected at 48 h post-transfection. Early apoptosis of K562 cells was detected with Annexin V-fluorescein isothiocyanate (FITC) and propidium iodide (PI) dual parameter flow cytometry. (A) Representative Annexin V/PI double staining and (B) comparison of the percentage of early apoptosis in each group. ^*^P<0.05 vs. the control and negative control (NC) groups. 1, Control; 2, NC; 3, PmiR-203; 4, arsenic trioxide (ATO); 5, ATO+NC; 6, PmiR-203+ATO.

PmiR-203 was used alone or in combination with ATO in K562 cells. Apoptosis of the K562 cells was detected by flow cytometry using double-staining with Annexin V and PI. The results demonstrated that PmiR-203 alone induces cell apoptosis and promotes the ATO-induced apoptosis of cells as compared with controls (Fig. 6).

### Detection of caspase-3 and caspase-9 activities using a colorimetric method

The caspase family plays an important role in the mediation of apoptotic progress and caspase-3 and caspase-9 are considered key performing elements in the process of apoptosis. The activities of these proteins are associated with DNA fragmentation, chromatin condensation and apoptotic body formation. Under normal conditions, caspase-3 and caspase-9 exist in the cytoplasm in the inactive pro-enzyme form; however, during apoptosis, they are activated and split the corresponding substrate in the cytoplasm and nucleus, finally leading to apoptosis. The activities of caspase-3 and caspase-9 were significantly increased in the PmiR-203, ATO and PmiR-203+ATO groups, indicating that PmiR-203 and ATO activate caspase-3 and caspase-9 (Table I).

### PmiR-203 downregulates the bcr/abl mRNA level as detected by real-time PCR

**Figure 7:**
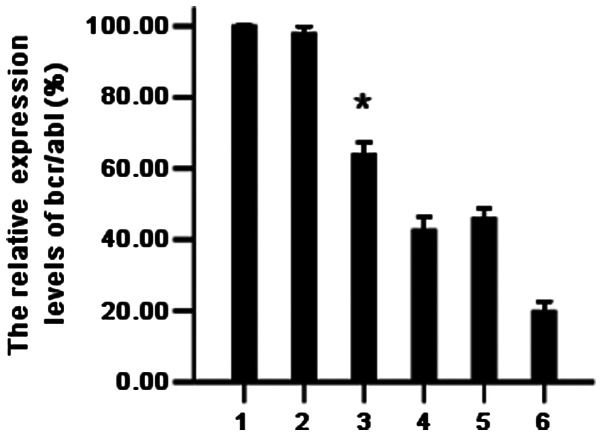
PmiR-203 regulated the bcr/abl mRNA level. Total RNA from treated K562 cells was extracted using TRIzol and quantified by ultraviolet spectrophotometry at 48 h after transfection. The bcr/abl mRNA relative expression level was assessed by SYBR-Green real-time polymerase chain reaction (PCR). The data demonstrated that PmiR-203 downregulates bcr/abl mRNA expression in K562 cells. ^*^P<0.01 compared with the control and negative control (NC) groups. 1, Control; 2, NC; 3, PmiR-203; 4, arsenic trioxide (ATO); 5, ATO+NC; 6, PmiR-203+ATO.

To evaluate the effects of PmiR-203 on the bcr/abl mRNA level of the cells, K562 cells transfected with PmiR-203 were processed and analyzed for mRNAs. The bcr/abl expression was determined by quantitative real-time PCR. GAPDH was used as the internal control. The fold-change of the expression level was calculated using 2^−ΔΔCt^, as described in Materials and methods. The 2^−ΔΔCt^ value of K562 cells treated with PmiR-203 was only 0.189 (18.9%). The results indicate that PmiR-203 effectively downregulates the bcr/abl level (Fig. 7).

### Release of cytochrome c protein detected by western blotting

**Figure 8:**
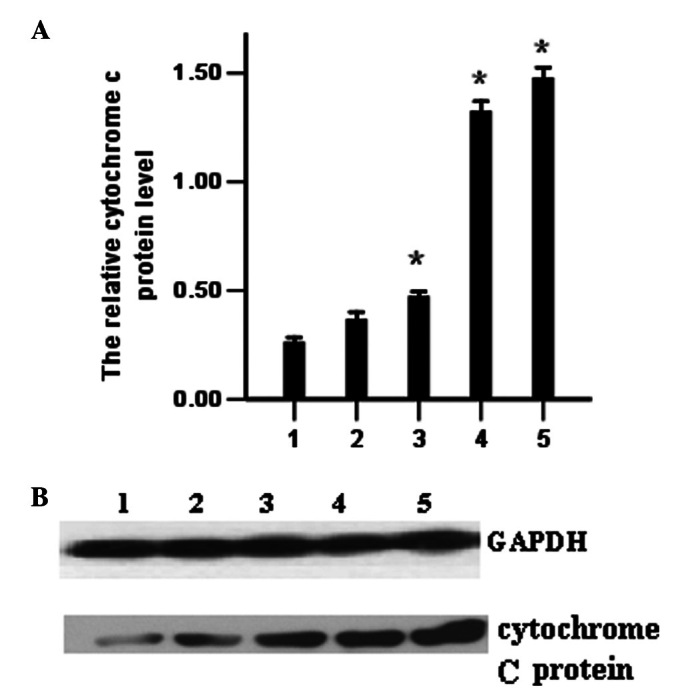
Cytochrome *c* protein levels increased. The cells were lysed at 48 h after transfection. The protein concentration was determined using bicinchoninic acid (BCA). Cytochrome *c* protein levels were determined by western blotting. (A) The relative cytochrome c protein level was increased (^*^P<0.01) as compared with that in the control and negative control (NC) groups. 1, Control; 2, NC; 3, PmiR-203; 4, arsenic trioxide (ATO); 5, ATO+NC; 6, PmiR-203+ATO. (B) Representative images of cytochrome *c* assessed by western blotting. GAPDH, glyceraldehyde 3-phosphate dehydrogenase.

To evaluate the levels of cytochrome *c* protein, K562 cells transfected with PmiR-203 were processed and analyzed for cytochrome *c* protein by western blotting as described in Materials and methods. The results indicate that the release of cytochrome *c* protein increased in the PmiR-203 and the ATO groups (Fig. 8).

## Discussion

Increasing evidence suggests that significantly low-expressed miRNAs in tumors may be considered to be tumor suppressor genes. These miRNAs usually suppress tumor development by negatively regulating oncogenes that control biological processes. Therefore, increasing the expression of tumor suppressor genes may be a valuable strategy for cancer treatment (9).

A number of studies have shown that miR-203 plays a role in carcinogenesis. Melar-New and Laimins (10) reported that high levels of miR-203 inhibit human papillomavirus (HPV) amplification and miR-203 has been proposed to be a new prognostic marker for pancreatic adenocarcinoma (11). miR-203 has also been proposed to be a tumor-suppressive miRNA in hepatocellular carcinoma and hematopoietic malignancies (7,12). In a previous study, we successfully constructed a eukaryotic expression vector expressing the hsa-miR-203 plasmid (PmiR-203) and then transfected it into K562 cells using Lipofectamine 2000. miR-203 presented a gain-of-function phenotype in K562 cells, which inhibits the proliferation of K562 cells (8).

The identification of target genes is a key step in assessing the role of aberrantly expressed miRNA in human cancer and is used for the further development of miRNA-based gene therapy. Yi *et al*(12) confirmed p63 as a target of miR-203. The authors demonstrated that miR-203 expression is conspicuous in terminally differentiating epithelial cells. abl1 is activated in hematopoietic malignancies in certain cases as a bcr-abl1 fusion protein (Philadelphia chromosome). bcr-abl1 fusion is a landmark of CML (13). The current study indicated that the bcr/abl mRNA expression level was downregulated by PmiR-203 (Fig. 7) and the results demonstrated that increasing the expression of PmiR-203 specifically reduces the expression of the reporter gene that carries two tandem bcr/abl-3′UTRs without changing the expression level of the mutations’ reporter gene in the seed sites (Fig. 1). The results demonstrate that bcr/abl is directly targeted by miR-203 in K562 cells.

The current study also indicated that ATO alone inhibits cell growth, increases the G1 phase population and induces early apoptosis in K562 cells (Figs. 2, 5 and 6). This is consistent with the study by Li *et al*(9). It has been reported that cytochrome *c* release from the mitochondria precedes dissipation of the voltage gradient (mitochondrial trans-membrane potential) across the inner membrane, which supports the specific channel hypothesis, suggesting that the escape of cytochrome *c* from the mitochondria occurs prior to permeability transition pore opening (loss of mitochondrial transmembrane potential) (14). In the current study, we demonstrated that ATO and PmiR-203 induce apoptosis of K562 cells. The induction of apoptosis may involve the loss of mitochondrial transmembrane potential, cytochrome *c* release and caspase-9 and caspase-3 activation, as shown in Figs. 3 and 8 and Table I. PmiR-203 significantly sensitized K562 cells to ATO by inducing apoptosis. When PmiR-203 and ATO were used in combination, PmiR-203 reduced the therapeutic dose of ATO; this may lead to low non-specific effects and high tolerance and also possibly reverse drug resistance in leukemia. Theoretically, this strategy may be feasible. Therefore, exploiting the synergistic effects between PmiR-203 and ATO may be an effective clinical strategy for leukemia chemotherapy and miR-203 may be a potential drug target in K562 cells.

## Figures and Tables

**Table I:** Activities of caspase-3 and caspase-9 (n=3).

Group	Caspase-3	Caspase-9
Control	0.103±0.02	0.105±0.01
NC	0.216±0.01	0.260±0.03
PmiR-203	0.452±0.01^a^	0.490±0.09^a^
ATO	0.434±0.03^a^	0.484±0.04^a^
ATO+PmiR-203	0.623±0.05^a^	0.636±0.06^a^
ATO+NC	0.412±0.02^a^	0.425±0.07^a^

Data are presented as mean ± standard deviation.

a P<0.01, compared with the control and NC groups. NC, negative control; ATO, arsenic trioxide.
